# Integrated airborne gamma-ray spectrometry, aeromagnetic analysis, and radiological assessment of sedimentary rocks, West Qasr El-Farafra area, Egypt

**DOI:** 10.1038/s41598-026-58831-1

**Published:** 2026-06-23

**Authors:** Ali M. El-Hawary, Hussein F. Abd El Salam, Reham M. Abd El Rhman, Mohamed Y. Hanfi

**Affiliations:** 1 Nuclear Materials Authority, El-Maadi, P.O. Box 530, Cairo, Egypt; 2 Department of Life Safety, Institute of Fundamental Education, Ural Federal University, Ekaterinburg, 620002 Russia; 3 Department of Physics, Dogus University, Dudullu-Ümraniye, 34775 Istanbul, Türkiye

**Keywords:** Airborne gamma spectrometry, Radionuclides, Sedimentary rocks, Statistical analysis, Environmental sciences, Solid Earth sciences

## Abstract

West Qasr El-Farafra area is particularly important for establishing new integrated urban communities. It is characterized by its flat area covered with slopes of limestone and sandstone limestone and sandstone rocks. Airborne gamma ray spectrometry and magnetic data were used for environmental radiation monitoring and mapping the basement surface in the west Farafra area. This study investigates the concentrations and distribution patterns of three radionuclides-^238^U, ^232^Th, and ^40^K-in sedimentary rocks. Analysis reveals that ^238^U has a mean concentration of 37 Bq kg^-1^ with high variability and a positively skewed distribution, suggesting localized enrichment processes. ^232^Th shows a mean concentration of 32 Bq kg^-1^ and exhibits moderate skewness and a flatter kurtosis, likely due to the presence of thorium-bearing minerals. In contrast, ^40^K has a mean concentration of 665 Bq kg^-1^ with the least variability and a near-normal distribution, attributed to the widespread presence of potassium-bearing minerals. Radiological parameters, including the radium equivalent activity (Ra_eq_), dose rate (D_air_), and annual effective dose (AED), generally indicate lower radiological hazards compared to global averages, though some samples exhibit elevated levels. Pearson correlation analysis demonstrates strong associations among the radionuclides and radiological risk indicators, with ^232^Th showing the highest correlation. Principal Component Analysis (PCA) and Hierarchical Cluster Analysis (HCA) further reveal that ^40^K and ^232^Th are more closely related, while ^238^U behaves distinctly, indicating different geological or environmental influences. Consequently, as a result of the above findings, the location of interest is generally radiologically acceptable for redevelopment purposes pending verification of a site specific nature within those areas where anomalous levels can be found.

## Introduction

Natural radionuclides have been present in the Earth’s crust since the planet’s birth and are a dynamic reservoir. The three most common of these elements ^238^U, ^232^Th, and ^40^K continue to undergo radioactive decay and emit ionizing radiation^1–3^. Even though these elements are observed in sedimentary rocks in numerous geological formations, their presence has been described a lot of great relevance by the scientific society and the public health authorities. While sedimentary rocks cover approximately 75% of the Earth’s surface, their significance proceeds elsewhere simple geology. These rocks serve as the substance for a diversity of ecosystems and scenes and sustain essential assets involving fossil fuels, groundwater, and construction materials. However, their ability to concentrate naturally occurring radionuclides creates unique health and environmental challenges that require careful consideration^4–6^.

Mineral and organic particles accumulate and lithify over millions of years to form sedimentary rocks. Radionuclides are often selectively enriched by processes including weathering, erosion, and deposition, especially in organic-rich shales, phosphorites, and certain sandstones^7–10^. For instance, uranium has been observed to bond with organic matter or phosphate minerals, but thorium has been found to mix with clay-rich sediments. This geological segregation gives rise to the pattern of specific high-radiation zones that may remain dormant until they are disturbed by natural erosion or by human operations like mining, construction, or hydraulic fracturing. The interchangeability of sedimentary rock lithology, geochemical conditions, and upward mobility of radionuclides highlights the difficulties in predicting and justifying the associated risks^11–13^.

There are many risks associated with the natural presence of radionuclides in sedimentary rocks. In the case of leakage of uranium-containing shales or sandstones, radon (Rn-222), a carcinogenic gas formed during the decay of uranium, enters the structure, which can lead to indoor air pollution and increase the risk of lung cancer. Moreover, it has been shown that using materials originating from sedimentary rocks, such as granite, sandstone, and clay bricks, in construction raises the levels of ambient radiation in homes and workplaces. The environmental and public health risks are exacerbated by radioactive uptake by crops in agricultural soil or their leaching into groundwater systems, in addition to direct exposure. In areas with high levels of background natural radiation or where industrial enterprises disrupt geological stability, these risks are often exacerbated^5,14,15^.

To cope with population and economic growth and to sustain land sustainably, we need to build new urban communities. One of the priorities in modern urban planning is to integrate residential, commercial, and industrial areas while preserving the environment. The shape of new urban areas is largely determined by the development of infrastructure, such as public services, utilities, and transport networks. Smart city solutions and advanced technologies are being increasingly introduced to enhance resource management, reduce environmental pollution, and boost energy efficiency. It has been proven that the targeted growth of urban areas benefits the local population by improving living standards, expanding access to employment opportunities and strengthening social cohesion^16,17^. The creation of new urban communities requires important growing strategies that consider various aspects such as ecology, economy and geography. Green building technologies, effective waste management approaches, and renewable energy bases form the basis of environmental urban development. Inhabitants need to be involved in the construction of urban communities. Large-scale initiatives aimed at meeting housing, transportation and other needs are funded in partnership between Governments and private investors. In newly built megacities, the integration of modern infrastructure with natural spaces and cultural centers can contribute to economic growth while preserving the integrity of the environment^18,19^.

The Western Qasr El-Farafra region, located in the Western Desert of Egypt, has attracted a lot of attention because of its potential to create innovative integrated urban settlements. Most of the relief of the region consists of flat landscapes with limestone and sandstone formations. To ensure environmental safety and public health, we estimate the levels of natural radiation^20,21^. Additionally, aeromagnetic data can be used to determine the depth of the aquifer, the surface of underlying rocks, the density of sediments, and shallow water levels^22^.

The study provides a regional-level overview of where radioactivity comes from in the West Al-Qasr Al-Farafra Region. A comprehensive study was done using 11,435 datasets collected with an airborne gamma-ray spectrometer to create a baseline of average natural radiation levels in the region and to identify areas where radiation could be a threat. The use of radiobiological hazard assessment combined with multivariate statistical methods, such as PCA (Principal Component Analysis) and HCA (Hierarchical Cluster Analysis), was used to understand how radioactive materials are linked together and what the public’s radiation exposure is due to them. This investigation offers a practical way for planners and decision-makers to include scientific data about radiation exposure in their plans and decisions, which promotes safe land management and protects the well-being of individuals, especially in arid land areas. The main objectives of the study are to assess the concentrations and distribution patterns of radionuclides of sedimentary rocks, calculate radiation effects and doses, and identify any health risks. The purpose of the study is to assess the suitability of the area for sustainable urban and agricultural development, as well as to assess the depth of the bedrock surface using two-dimensional magnetic modelling.

## Geological setting

**Fig. 1:**
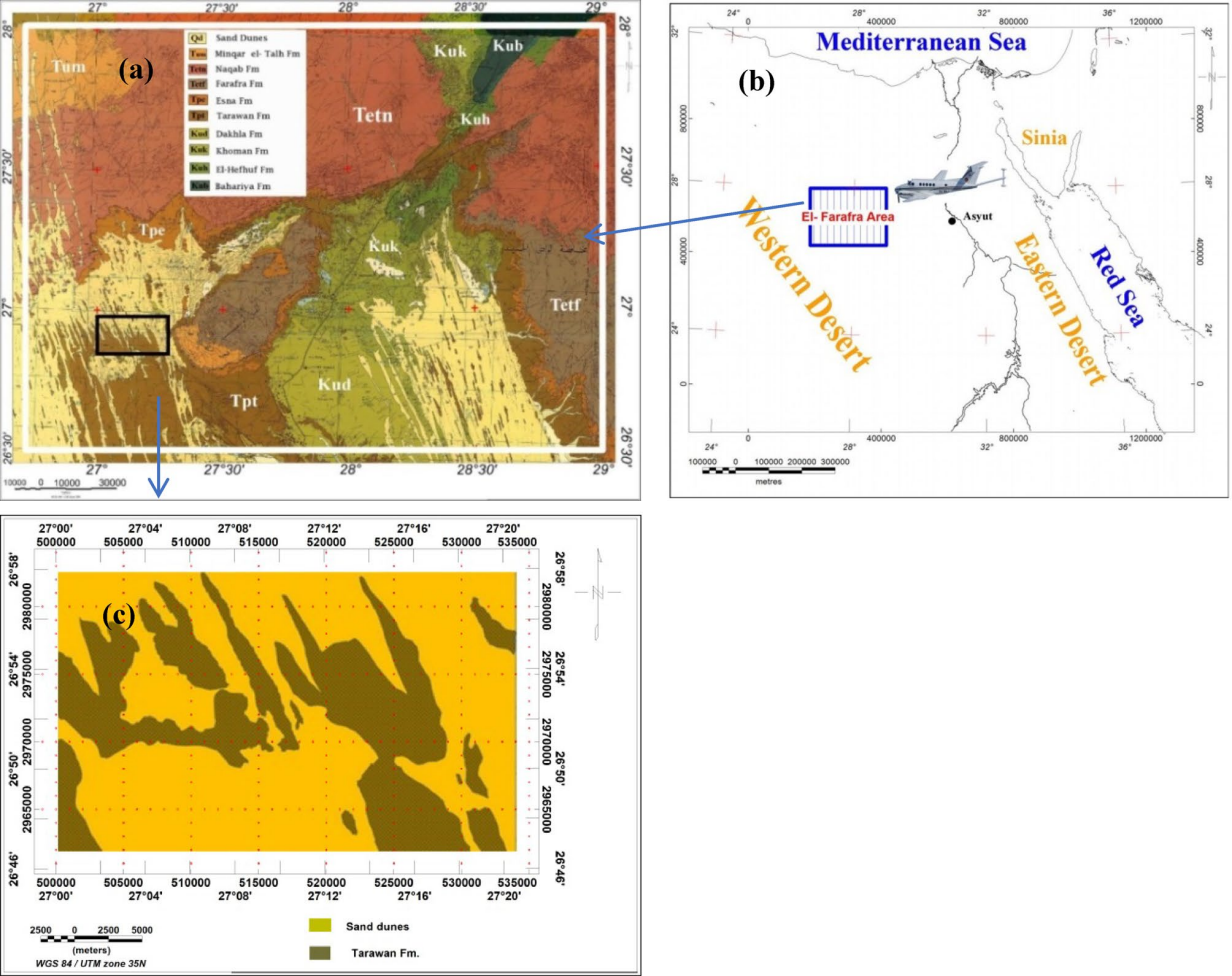
(**a**) Location map Farafra area, (**b**) Geologic map of the Farafra area, (after Conoco, 1987), (**c**) Geologic map of the west Qasr El-Farafra area, Central Western Desert, Egypt.

The early contributions of prominent scientists, such as^23,24^ mark the history of geological research in the Farafra region. Then came an intensive period from^25^ to^26 and 27^. The region’s geologic framework is delineated in a map published by Conoco Coral (1987) (Fig. 1), which reveals the presence of sedimentary rocks spanning the Late Cretaceous (Campanian) to Eocene, alongside Quaternary NW-SE-oriented sand dunes. Hermina (1990) unified the stratigraphy into the Campanian–Lower Eocene marine deposits series, a group encompassing diverse marine and transitional facies^26^. This sequence, characterized by alternating clastic and carbonate deposition associated with marine transgression and regression, provides a fundamental understanding of the geological evolution of the area. The Campanian–Early Eocene sequence begins with the Wadi Hennis Formation (Early–Middle Campanian), comprising clays and sandstones unconformably overlain by the carbonate-rich El Hefhuf Formation (Upper Campanian). The latter, a dolomite and chalk unit devoid of clastics, correlates with the Duwi Formation and contains Upper Campanian ammonites. The overlying Khoman Formation (Maastrichtian) consists of white chalky calcilutite, exposed thinly near Qasr El Farafra. The Cretaceous sequence culminates in the Dakhla Formation (Maastrichtian), characterized by clayey shales indicative of shallow marine conditions, which underlie Palaeocene carbonates. Post-Cretaceous marine transgression deposited Palaeocene units, including the Kurkur Formation (reefal limestone with clay interbeds) and Garra Formation (shallow marine limestone), marking a shift to carbonate-dominated sedimentation. The Early–Middle Eocene Farafra Formation represents a platform facies of the Thebes Group, transitioning laterally into chert-rich facies (El Rufuf Formation) and forming a 200-km outcrop belt. Concurrently, the Naqb Formation (Early–Middle Eocene) unconformably caps Palaeocene chalk north of the Farafra depression, reflecting regional uplift that suppressed Esna Formation deposition.

Lithological composition and depositional environment significantly affect how the natural radionuclides are distributed across all of the sedimentary successions being analysed. The clay-dominated shale components of the Dakhla and Tarawan formations have the potential to lead to uranium and thorium enrichment via adsorption onto clay minerals, accumulation of organic materials, and via phosphate related mineral formation. Sandstone units typically provide lower concentrations of radionuclide elements because of the generally lower amounts of clay and accessory minerals in sandstone. In the case of sandstone, localized uranium enrichment may occur via processes that lead to secondary mobilization and deposition of uranium in areas where it is found in elevated concentrations in the sandstones. In carbonate formations, such as limestone and chalky units, the radionuclide concentrations for ^238^U, ^232^Th and ^40^K are also typically lower due to dilution caused by the carbonate minerals. Potassium concentrations are found in the sediments that contain feldspar and clay; therefore, there is a relatively widespread occurrence of ^40^K throughout the sedimentary sequence.

## Materials and methods

### Aerial geophysical survey data

A high-resolution aerial magnetic and spectrometry measurement was conducted over the study area (West El-Farafra Area) by the airborne geophysics Department, Exploration Division of the Nuclear Material Authority (NMA) of Egypt. The geophysical data were measured along the designed equally spaced parallel traverse lines spaced at 2 Km. The direction of the traverse lines was selected at a directions of (0° N). The tie lines were spaced at 9000 m perpendicular to the traverse lines at a directions of (90° E). The terrain clearance adopted for the aircraft during normal survey flying was 100 m above the ground surface. The normal aircraft average air speed was around 250 km/h.

Unlike laboratory tests on gathered rock samples, the radionuclide data set utilized in this research study (*N* = 11435) was collected through airborne gamma-ray spectrometry. This airborne study yields continuous spatial representations of equivalent uranium (eU), equivalent thorium (eTh), and potassium (K) concentrations at a surface location without interruption. The onboarding/ground support team followed the industry norms for data processing and quality control so that accurate measurements could be attained (for example: removing background and air craft noise, cosmic radiation was corrected via upward looking detector, altitude was normalized to nominal flight height, spectral stripping separated contributions of U, Th, and K, while using the calibration protocol from Nuclear Material Authority (NMA) for calibrations). The estimated uncertainty with respect to these airborne measurements varies from approximately 5% to 10%, based on a variety of criteria such as flight altitude, terrain conditions, counting statistics, and radionuclide concentration levels.

After corrections are made, all data from a specific gamma-ray survey should be correlated to a single baseline gamma-ray count that represents a value for all surviving gamma-ray counts. The radiometric anomalies compared with mapped lithological units demonstrate that the airborne radionuclide concentration values show wider geographic and spatial patterns than previously filed, hence providing indirect validation that the derived airborne radionuclide concentrations from airborne radiometric studies are indeed accurate. For airborne gamma-ray spectrometry, altitude calibration is a significant correction item that helps correct for the effects of atmospheric attenuation, which reduces the intensity of measured gammas. Gamma-rays acquired along with the survey have been recorded in real time based on the elevations of the aircraft above the terrain and were later normalized to a fixed reference altitude with the use of altitude attenuation coefficients as determined during calibration. By using this correction for altitude, the effects of aircraft altitude change can be completely removed, meaning that differences in gamma count rates can only be attributed to changes in the amount of radioactivity present at that location and not to the acquisition angle.

The Picoda (PGAM-1000) is a gamma-ray spectrometer that is characterized by a 256-channel sensitive system that is installed in the aircraft. The device has a spectral data range from 0 to 3 MeV and is characterized by its self-stabilization mechanism, which is designed to minimize spectral drift. The detector package includes three crystal boxes (A, B, C), with a total volume of 3,328 cubic inches (54.535 L). The detector package contains two detectors: the first detector is used to detect terrestrial gamma radiation (the downward-facing detector), while the second detector is used to detect cosmic and atmospheric radiation (the upward-facing detector). The configuration of the first detector comprises 12 high-resolution crystals of thallium-activated sodium iodide NaI (Tl), each accompanied by its own photomultiplier tube (PMT). The configuration under consideration produces a total volume of 3,072 cubic inches (50.34 L). The second detector, designed as an upward-pointing detector, consists of a high-precision crystal of thallium-doped sodium iodide NaI(Tl), with a total volume of 256 cubic inches (4.195 L). The spectrometer is designed to meticulously monitor a prominent photopeak, thereby enabling the automatic adjustment of gains for photomultiplier tubes for each crystal. This adjustment is pivotal in ensuring the spectral stability of the system. Furthermore, the spectrometer is equipped with three dedicated windows for the purpose of monitoring gamma rays. These windows are set to specific energies, including 1460 keV for ^40^K, 1760 keV for ^214^Bi from the uranium decay series, and 2615 keV for ^208^Tl from the thorium decay series. A total count window is utilized to monitor overall radioactivity levels. The detector count rates are susceptible to the influence of cosmic radiation, which exhibits an upward trend with increasing altitude above sea level. To track this increase, a cosmic ray window records all incident particles above 3 MeV^28^. Total counts were measured in (T.C., µR/h), equivalent uranium (eU, ppm), equivalent thorium (eTh, ppm), and potassium (K, %). The use of the red-blue-green (RBG) color scheme proved useful in producing various composite image maps. These maps were digitized using the Oasis Montaj software. Airborne magnetometer: A Scintrex MMS-4 airborne Cesium magnetometer system was used during the survey. This system utilizes a split-beam, optically pumped Cesium vapor magnetic sensor, having a sensitivity of $\:0.001\:nT$, and a sampling rate of 0.1 s. The sensor capabilities guaranteed correct sampling of high magnetic gradient zones. The total field intensity of this instrument is approximately ranges from 15,000 to 105,000 nT.

According to the following equation, radionuclides were evaluated for activity concentrations:

1 $${\text{A }}\left( {{\text{Bq k}}{{\mathrm{g}}^{ - {\mathrm{1}}}}} \right){\text{ }} = {\text{ }}{{\mathrm{C}}_{\mathrm{i}}} \times {\text{ CF}}$$

Where A is activity concentration of radionuclide (Bq kg^− 1^), C is concentration measured from airborne gamma ray spectrometric data (ppm for eU and eTh; % for K) and CF is the conversion factor. The conversion factors used were ^238^U-12.35 Bq kg^− 1^/ppm; ^232^Th-4.06 Bq kg^− 1^/ppm; and ^40^K-313 Bq kg^− 1^/%^29^.

The radiological parameters, including radium equivalent (Ra_eq_^30^, absorbed dose rate (D (nGy h^− 1^)^31,32^, annual effective dose (AED (mSv y^− 1^)^2,33^, and excess lifetime cancer risk (ELCR×10^− 3^^34^ are assessed according to the following equations:

2 $${\mathrm{R}}{{\mathrm{a}}_{{\mathrm{eq}}}}\left( {{\text{Bq k}}{{\mathrm{g}}^{ - {\mathrm{1}}}}} \right) = {\text{ }}{{\mathrm{A}}_{\mathrm{U}}} + {\text{ 1}}.{\text{43 }}{{\mathrm{A}}_{{\mathrm{Th}}}} + {\text{ }}0.0{\text{77 }}{{\mathrm{A}}_{\mathrm{K}}}$$

3 $${{\mathrm{D}}_{{\mathrm{air}}}}\left( {{\text{nGy }}{{\mathrm{h}}^{ - {\mathrm{1}}}}} \right)~ = {\text{ }}0.{\mathrm{43}}0{{\mathrm{A}}_{\mathrm{U}}} + 0.{\mathrm{666}}{{\mathrm{A}}_{{\mathrm{Th}}}} + {\text{ }}0.0{\mathrm{42}}{{\mathrm{A}}_{\mathrm{K}}}$$

4 $$\:{AED}_{out}\left(\frac{mSv}{y}\right)=\:\sum\:\left({D}_{air}\:\left(\frac{nGy}{h}\right)\times\:0.7\:\left(\frac{Sv}{Gy}\right)\times\:0.2\right)\times\:8760h\:\times\:{10}^{-6}\:\:\:$$

5 $$\:{AED}_{in}\left(\frac{mSv}{y}\right)=\:\sum\:\left({D}_{air}\:\left(\frac{nGy}{h}\right)\times\:0.7\:\left(\frac{Sv}{Gy}\right)\times\:0.8\right)\times\:8760h\:\times\:{10}^{-6}\:\:\:$$

D_air_ is the absorbed dose rate in air, expressed as nGy h^− 1^, with 8760 indicating the number of hours from one calendar year. The dose conversion coefficient between absorbed dose in air and effective dose is 0.7 Sv Gy^− 1^, while the outdoor and indoor occupancy factors are 0.2 and 0.8, respectively, per UNSCEAR recommendations.

Finally, the Excess Lifetime Cancer Risk (ELCR) was determined to estimate how likely someone could get cancer through their exposure to man-made sources of radioactive material over duration time (DL = 70 years). And the cancer risk factor is RF, 0.05 Sv^− 1^^32,35^.

6 $$\:{\mathrm{E}\mathrm{L}\mathrm{C}\mathrm{R}}_{out}=\:{\mathrm{A}\mathrm{E}\mathrm{D}}_{out}\:\mathrm{x}\:\mathrm{D}\mathrm{L}\:\mathrm{x}\:\mathrm{R}\mathrm{F}\:$$

7 $$\:{\mathrm{E}\mathrm{L}\mathrm{C}\mathrm{R}}_{in}=\:{\mathrm{A}\mathrm{E}\mathrm{D}}_{in}\:\mathrm{x}\:\mathrm{D}\mathrm{L}\:\mathrm{x}\:\mathrm{R}\mathrm{F}\:$$

8 $${\text{ELCR }} = {\text{ ELC}}{{\mathrm{R}}_{{\mathrm{out}}}} + {\text{ ELC}}{{\mathrm{R}}_{{\mathrm{in}}}}$$

To evaluate the excess lifetime cancer risk, the analysis was based on two different types of exposure scenarios; one for outdoor exposures and one for indoor exposures. Separate evaluations were necessary due to differences in exposure duration and occupants per unit area of outdoor vs. indoor spaces. ELCR for outdoor (ELCR_out_) is representative of an individual’s likelihood of developing cancer from extended outdoor exposure to terrestrial gamma radiation, whereas ELCR for indoor (ELCR_in_) is representative of the cancer likelihood experienced from being indoor. To derive a total ELCR, both outdoor and indoor ELCR were summed to give a single value representing the total calculated lifetime radiological (gamma radiation) risk to the public.

### Multivariate statistical analysis

The relationships between the concentrations of radionuclides (^238^U, ^232^Th and ^40^K) and their corresponding radiological hazard indices (estimation of the total hazard index Ra_eq_, D_air_, AED_out_, AED_in_, and ELCR) were studied using Multivariate statistical analysis techniques such as Principal Component Analysis (PCA) and Hierarchical Cluster Analysis (HCA). Prior to any analysis, all variables have been standardized using z scores to negate the effects of differing units and scales. The PCA was conducted using the correlation matrix as opposed to the covariance matrix, which ensures that higher variance variables do not dominate the analysis. All components with eigenvalues exceeding one were retained, and the component loadings were used to ascertain the contributions of each variable. The HCA analysis was based on the Euclidean distance as a measure of similarity and utilized Ward’s method of cluster linkage; Ward’s method minimizes the variances within clusters and is therefore most suited to environmental dataset analysis. As exploratory tools for identifying and highlighting the overall patterns and associations of radiological datasets, we used PCA and HCA as the primary analytical techniques.

## Results and discussion

### Radioactive content

**Fig. 2:**
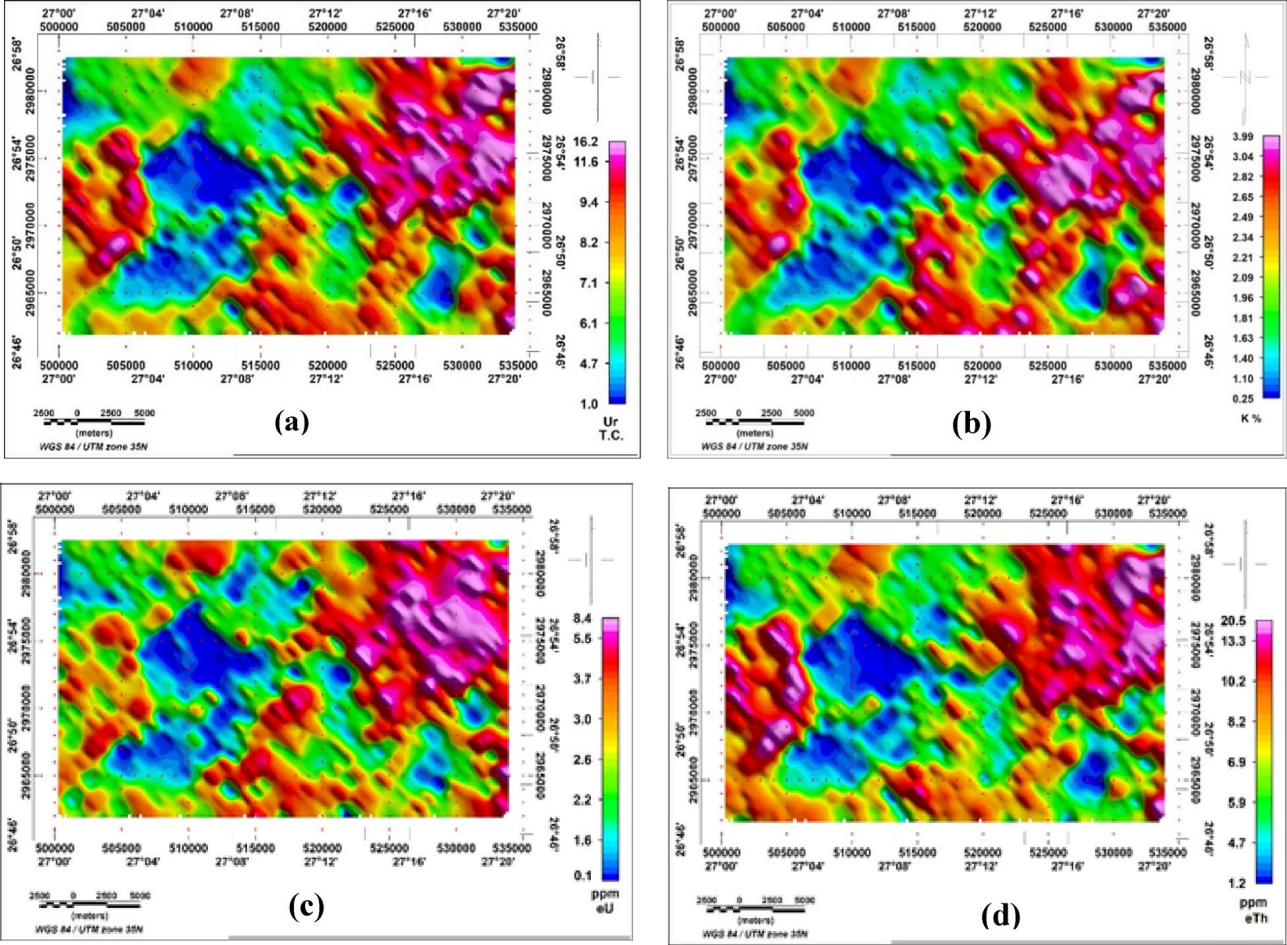
(**a**) Total count map (in Ur), (**b**) Potassium map (in %), (**c**) Equivalent uranium map (in ppm), (**d**) Equivalent thorium map (in ppm) at west Qasr El-Frafra area, central western desert, Egypt.

According to a detailed examination of the different radio-spectrometric maps (T.C., $\:\mathrm{e}\mathrm{U}$, $\:\mathrm{e}\mathrm{T}\mathrm{h}$, and $\:\mathrm{K}$), these four maps (Fig. 2a–d) are very similar in terms of the relief of general features, the gradient of contours, the distribution of anomalies, the trend of radiometric surficial structures, etc. The broad radiometric characteristics and levels for the various rock types in the research region were established by carefully examining the four maps, particularly the areas where the agreement is greatest. The distribution of the high radioelement concentrations is known to be substantially influenced by the Tarwan Formation next to the El-quss abu-said plateau, as can be seen by closely examining the total-count and the three-radioelement maps eU, eTh and K. In general, a fairly broad range of radioactivity is shown by the three radiometric maps (TC, eU and eTh) of the investigated region. These maps range from around 0.1 to 16.2, 0.1 to 8.4 ppm and from 1.2 to 20.5 ppm, respectively. These ranges show varying amounts of radioactivity inside and between the various rock types in the area, by agrees with the geologic map of the region (Fig. 1c). In general, three zones with overlapping radioactivity levels may be created from the three maps. The first is extremely low levels of radioactivity, which are mostly found in the southwest and west-central regions. The second is almost all area-based zones with relatively low radioactivity. The Tarwan Formation is linked to the third and comparatively modest radioactive zone. However, the potassium map displays a limited range of values (between 0.25 and 3.99%) over the research areas exposed rock units. Notwithstanding this small range, the three levels and the distribution of the radiation anomalies are in good accord with the earlier total-count, $\:\mathrm{e}\mathrm{U}$, and $\:\mathrm{e}\mathrm{T}\mathrm{h}$ maps.

### ^238^U, ^232^Th and ^40^K concentrations and their hazards

Table 1 shows that the analysis of the radionuclides ^238^U, ^232^Th, and ^40^K in sedimentary rocks reveals large differences in their concentrations and distribution patterns. ^238^U shows a mean concentration of 37 Bq kg^− 1^ with a wide range from 1 to 106 Bq kg^− 1^, reflecting a large variability, as shown by a high coefficient of variation (CV) of 50%. The distribution is skewed to the right, meaning that most samples have lower concentrations, but some have much higher ones. Higher values are at the ends of the distribution, forming a long tail. This difference indicates that the concentration of uranium in sedimentary rocks is affected by a combination of local enrichment processes and the different types of uranium-containing minerals present. ^232^Th, with a mean concentration of 32 Bq kg^− 1^, also shows significant variability from 5 to 85 Bq kg^− 1^, though slightly less than ^238^U, with a CV of 43%. The distribution is relatively skewed to the right, indicating that there are more samples with lower concentrations and fewer with higher values. The kurtosis of ^232^Th (-0.11) indicates a wider range of data about the mean. It could be attributed to the presence of thorium-bearing minerals, including monazite and zircon, which are more uniformly distributed in sedimentary areas than uranium. Otherwise, ^40^K reveals a significantly higher mean concentration of 665 Bq kg^− 1^ with a broader range from 82 to 1249 Bq kg^− 1^ and the lowest CV of 31%, indicating more reliable concentrations across the samples. The slight left-skewness and platykurtic distribution (flatter than usual) of ^40^K propose a more even distribution of potassium-bearing minerals such as feldspar and micas in sedimentary rocks. It can be seen in the studied samples the mean concentrations of ^238^U and ^232^Th are comparable and lower than the worldwide average of 35 and 45 Bq kg^− 1^, respectively^36^, respectively, as well as the mean concentration of ^40^K is 1.5 times higher than the worldwide average of 412 Bq kg^− 1^^36^. The higher concentrations of ^238^U, ^232^Th and ^40^K in sedimentary rocks may be due to several geological processes. Uranium and thorium are typically concentrated in heavy mineral deposits and can become enriched in sedimentary environments through processes like weathering and erosion of source rocks, followed by transportation and deposition in basins. Potassium, being a major element in common rock-forming minerals, naturally occurs in high concentrations. The variability in concentrations and distribution patterns among these radionuclides reflects the diverse mineralogical composition and the different geochemical behaviors of uranium, thorium, and potassium in sedimentary environments.

**Table 1:** Descriptive statistics of the studied sedimentary rocks of the studied area, Egypt.

	*N*	Mean	SD	Min	Max	Skewness	Kurtosis	CV, %
^238^U, Bq kg^− 1^	11,435	37	18	1	106	0.98	0.93	50
^232^Th, Bq kg^− 1^	11,435	32	14	5	85	0.60	-0.11	43
^40^K, Bq kg^− 1^	11,435	665	205	82	1249	-0.16	-0.57	31

**Fig. 3:**
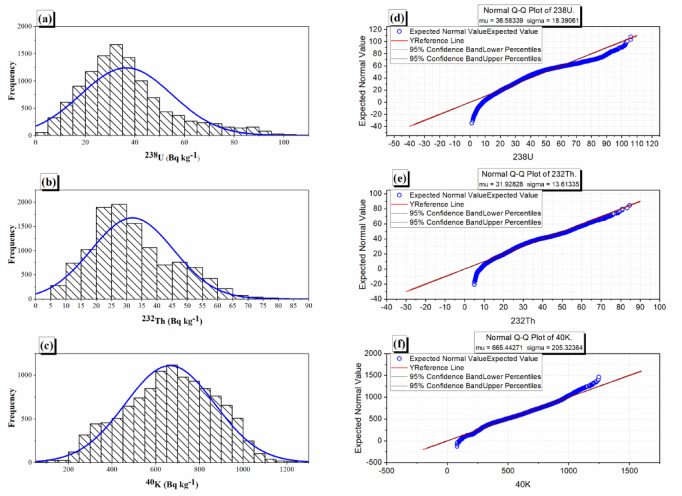
Frequency distribution histograms with fitted normal curves for (**a**) ^238^U, (**c**) ^232^Th, and (**e**) ^40^K activity concentrations and Normal Q–Q plots for (**b**) ^238^U, (**d**) ^232^Th, and (**f**) ^40^K activity concentrations of the sedimentary rocks in the studied area.

The provided Fig. 3 contains two types of plots for each of the three radionuclides ^238^U, ^232^Th, and ^40^K: frequency distribution histograms with fitted normal curves and Normal Q-Q (quantile-quantile) plots. The distribution and Q-Q plots of the radionuclides ^238^U, ^232^Th, and ^40^K in sedimentary rocks reveal distinct patterns in their concentrations. The histograms show that ^238^U has a positively skewed distribution, with most samples having lower concentrations and a few exhibiting much higher values. This skewness is confirmed by the Q-Q plot, where the points deviate significantly from the expected normal distribution, particularly in the tails, indicating that ^238^U concentrations are not normally distributed. For ^232^Th, the distribution is slightly less skewed compared to ^238^U, with a more centered concentration around the mean, though it still shows some deviation from normality. The Q-Q plot of ^232^Th reflects this moderate skewness, with points beginning to diverge from the normal line at higher concentrations, suggesting that while the data is closer to a normal distribution than ^238^U, it still isn’t perfectly normal. Although ^40^K looks like it is normally distributed based on visual analysis of the data, and it also fits a normal distribution based on the histogram and Q-Q plot; however, the Kolmogorov-Smirnov test indicates that it is not significantly different from normal. This suggests that ^40^K is more uniformly distributed compared to ^238^U and ^232^Th, which may reflect different geological processes or sources affecting the distribution of these radionuclides.

Table 2 presents the results of a Kolmogorov-Smirnov test applied to three radionuclides in the sedimentary rocks: ^238^U, ^232^Th, and ^40^K, with a large sample size of 11,435 degrees of freedom for each radionuclide. The Kolmogorov-Smirnov statistic, which measures the deviation of the observed data from the expected distribution, varies among the radionuclides, with ^238^U showing the highest deviation (0.09), followed by ^232^Th (0.08), and ^40^K (0.03). The p-values (Asymptotic Significance) associated with these statistics are extremely small, all far below the common significance threshold of 0.05 (e.g., 9.28E-90 for ^238^U. These low p-values indicate that the differences between the observed and expected distributions are statistically significant, leading to the rejection of the null hypothesis that the data follows a normal distribution. In summary, the test results suggest that the distributions of ^238^U, ^232^Th, and ^40^K in the dataset significantly deviate from normality.

**Table 2:** Results of normality tests.

Radionuclide	Kolmogorov-Smirnov*		
	DF	Statistic	Asymp. Sig. (2 tail)
^238^U	11,435	0.09	9.28E-90
^232^Th	11,435	0.08	1.99E-57
^40^K	11,435	0.03	3.92E-07

Asymp. sig. = asymptotic significance level. DF = degrees of freedom. *Lilliefors significance correction.

The statistical results from the Kolmogorov-Smirnov test, which indicated significant differences from univariate normality, are expected due to the very large number of observations in the current study (*N* = 11,435). Even small differences from a normal distribution can have very high p-values, so descriptive statistics, histograms and Q-Q plots were used to support the interpretation of distributional properties.

**Fig. 4:**
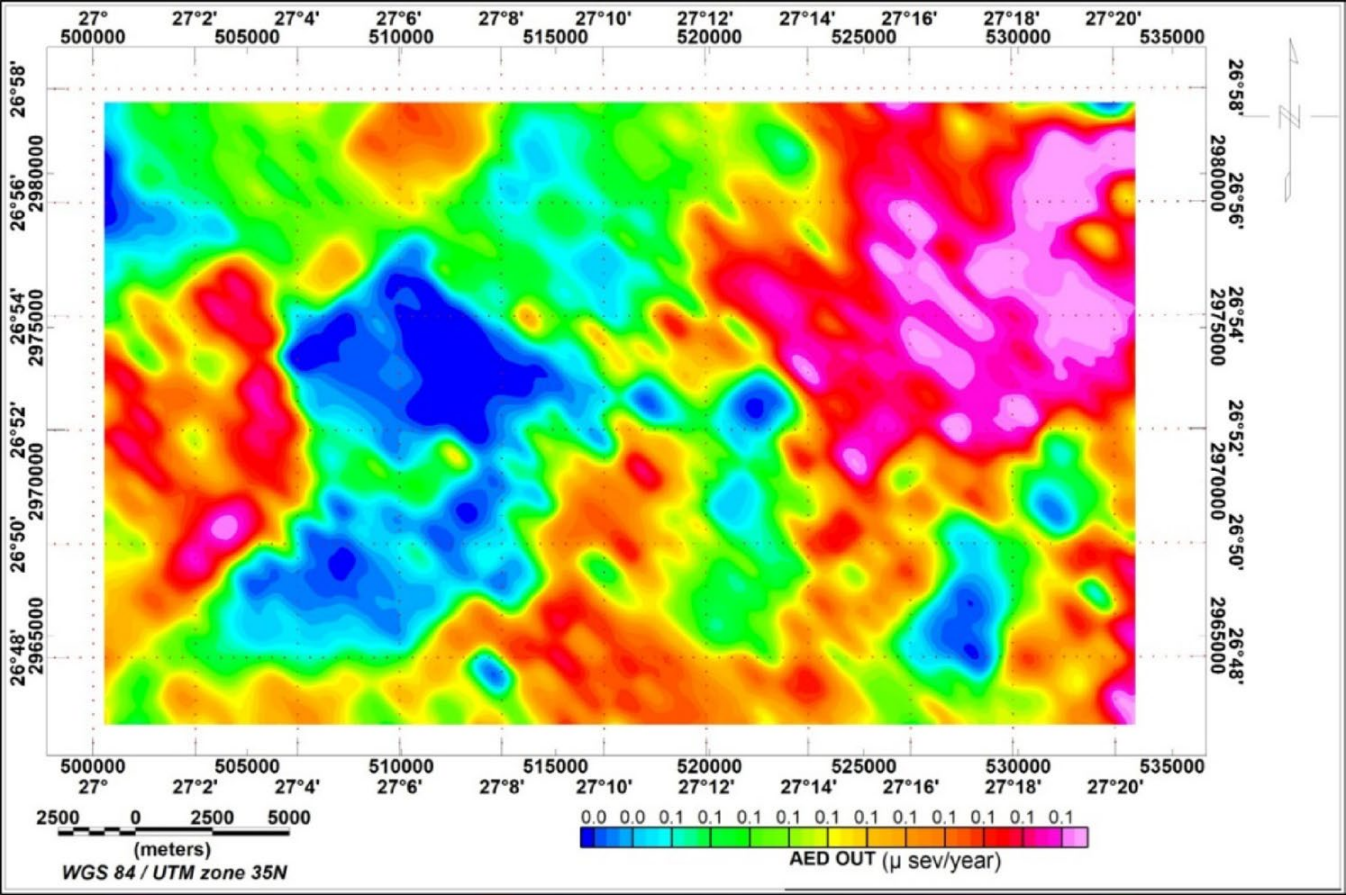
Effective outdoor dose map, west Qasr El Farah area. Central Western Desert, Egypt.

Table 3 illustrates that the analysis of radiological parameters in sedimentary rocks, on average, the Ra_eq_ is 133 Bq kg^− 1,^ with a range between 18 and 295 Bq kg^− 1^, which is significantly lower than the worldwide average of 370 Bq kg^− 1^^2^. This suggests that these rocks generally pose a lower radiological hazard compared to global levels. The D_air_ is ranged from a minimum of 8 to a maximum of 137 nGy h^− 1^ with the mean of 63 nGy h^− 1^, slightly above the global average of 59 nGy h^− 1^^1^, indicating marginally elevated environmental radiation from these rocks. Although the majority of the samples have a dose rate near this average, some reach as high as 137 nGy h^− 1^, which could be of concern in localized areas. In terms of radiation exposure, the outdoor annual effective dose (AED_out_) varies from 0.01 to 0.17 mSv y^− 1^ with a mean of 0.08 mSv y^− 1^, just above the global average of 0.07 mSv y^− 1^^1^, indicating that radiation exposure from these rocks in outdoor settings is generally comparable to global norms. However, the indoor annual effective dose (AED_in_) values range from 0.04 mSv y^− 1^ to 0.67 mSv y^− 1^, with an average of 0.31 mSv y^− 1^, which is below the global average of 0.41 mSv y^− 1^^37^, suggesting lower indoor radiation exposure from these rocks overall. Despite this, some samples show higher AED_in_ values, potentially increasing indoor radiation risks. Overall, while these sedimentary rocks are generally safe, the presence of some samples with elevated radiological parameters indicates a need for careful monitoring and management, particularly in areas where higher values are detected, as shown in (Fig. 4).

The Excess Lifetime Cancer Risk (ELCR) associated with these rocks averages 0.27 × 10^− 3^, slightly below the worldwide average of 0.29 × 10^− 3^^34,38^, implying a similar or slightly lower lifetime cancer risk from radiation. While the range of ELCR_in_ values is 0.14 × 10^− 3^ to 2.35 × 10^− 3^ with an average of 1.09 × 10^− 3^. This average value is below the world mean value of 1.4 × 10^− 3^^34,38^ and is therefore within the acceptable limits for lifetime cancer risk from natural sources of radiation as based on the entire ELCR indicator. However, the average total ELCR is much higher than the expected value at this site, suggesting localized areas with higher-than-expected levels of natural radiation and therefore require more monitoring moving forward^38^.

**Table 3:** Radium equivalent activity (Ra_eq_), absorbed dose rate (D_air_), annual outdoor effective dose (AED_out_), annual indoor effective dose (AED_in_), and Excess lifetime cancer (ELCR) in the sedimentary rock samples of the studied area, Egypt.

	*N*	Mean	SD	Min	Max
Ra_eq_ (Bq kg^− 1^)	11,435	133	50	18	295
D_air_ (nGy h^− 1^)	11,435	63	23	8	137
AED_out_ (mSv y^− 1^)	11,435	0.08	0.03	0.01	0.17
AED_in_ (mSv y^− 1^)	11,435	0.31	0.11	0.04	0.67
ELCR_out_ (×10^− 3^)	11,435	0.27	0.10	0.04	0.59
ELCR_in_ (×10^− 3^)	11,435	1.09	0.40	0.14	2.35
ELCR (×10^− 3^)	11,435	1.36	0.50	0.18	2.94

Hazard indices for radiological hazards deliver vital data regarding whether or not the sedimentary rock types that were evaluated can be used for construction and environmental purposes. For example, the radium equivalent activity (Ra_eq_) has been the most widely accepted representative of the sum of the radiological effects due to ^238^U, ^232^Th and ^40^K, based on a single index. Our mean Raeq value of 133 Bq kg^− 1^ is significantly below 370 Bq kg^− 1^ (the internationally acceptable limit) and therefore indicates that the evaluated rock materials pose very little to no gamma radiation risk to the general public. The D_air_ also reflects the amount of externally received gamma radiation resulting from the natural radionuclides occurring in the studied area. Although the average Dair is slightly above the global average for similar sampled data, and is still within acceptable environmental limits, the data indicate that the potential radiological impact of the natural radionuclides at the sites studied is relatively low. With respect to annual cumulative effective doses for outdoor and indoor exposures, the results show that virtually all of the locations represented within the sample set are reasonably safe from radiological hazards created by human activity, and therefore would be acceptable for continued development by the urban environment. In addition, findings indicate that ELCR values provide evidence that the risk of developing long-term radiological health problems for the affected population is very low; however, localized areas with elevated radiological values will require continued monitoring of their environment over time until approved large-scale development project can occur.

The observed Ra_eq_, D_air_, AED_out_, AED_in_, and ELCR values vary depending on how much ^238^U, ^232^Th, and ^40^K are present in the sedimentary formations where they were measured. High indices correspond to sedimentary formations enriched with uranium- or thorium-bearing minerals such as the Tarawan Formation, or those located in sedimentary formations associated with specific structural features. While the average values are generally below the internationally accepted level, the presence of localized anomalies makes it essential to do site-specific evaluations before large urban or agricultural developments happen. Both the lithology of the area and subsurface structural conditions identified in the integrated geophysical data influence radiogenic materials in the study area.

Although radionuclide concentration distributions did not fit a normal distribution, Pearson correlation coefficients, which represent the relationship between two variables, provide reliable information on how closely related two variables are, and have been used in many environmental and radiation-related fields with similar types of data and provide consistent results across large datasets. Table 4 displays the Pearson correlation analysis reveals strong associations between the radionuclides ^238^U, ^232^Th, and ^40^K, indicating that these elements are likely influenced by similar factors or co-occur in the sedimentary rock samples. The correlations are particularly high between ^238^U, and ^232^Th (0.81), as well as between ^232^Th and ^40^K (0.82), suggesting a significant relationship among these radionuclides. Additionally, all three radionuclides show very strong positive correlations with the radiological risk indicators, such as Ra_eq_, D_air_, AED_out_, AED_in_, and ELCR, with correlation coefficients ranging from 0.91 to 0.95. Among these, ^232^Th has the highest correlations with the risk indicators (0.95), indicating its substantial influence on radiological hazards. ^238^U and ^40^K also contribute significantly to the radiological risks, as reflected by their strong correlations with the risk indicators. Overall, the consistency in these high correlation values across different risk indicators highlights the importance of these radionuclides in assessing the potential radiological impact of the material.

**Table 4:** Pearson correlation between natural radionuclides and the radiological hazard coefficients of sedimentary rocks at the studied area, Egypt.

	^238^U	^232^Th	^40^K	Ra_eq_	D_air_	AED_out_	AED_in_	ELCR
^238^U	1.00	0.81	0.74	0.92	0.92	0.92	0.92	0.92
^232^Th	\\	1.00	0.82	0.95	0.95	0.95	0.95	0.95
^40^K	\\	\\	1.00	0.91	0.92	0.92	0.92	0.92

**Fig. 5:**
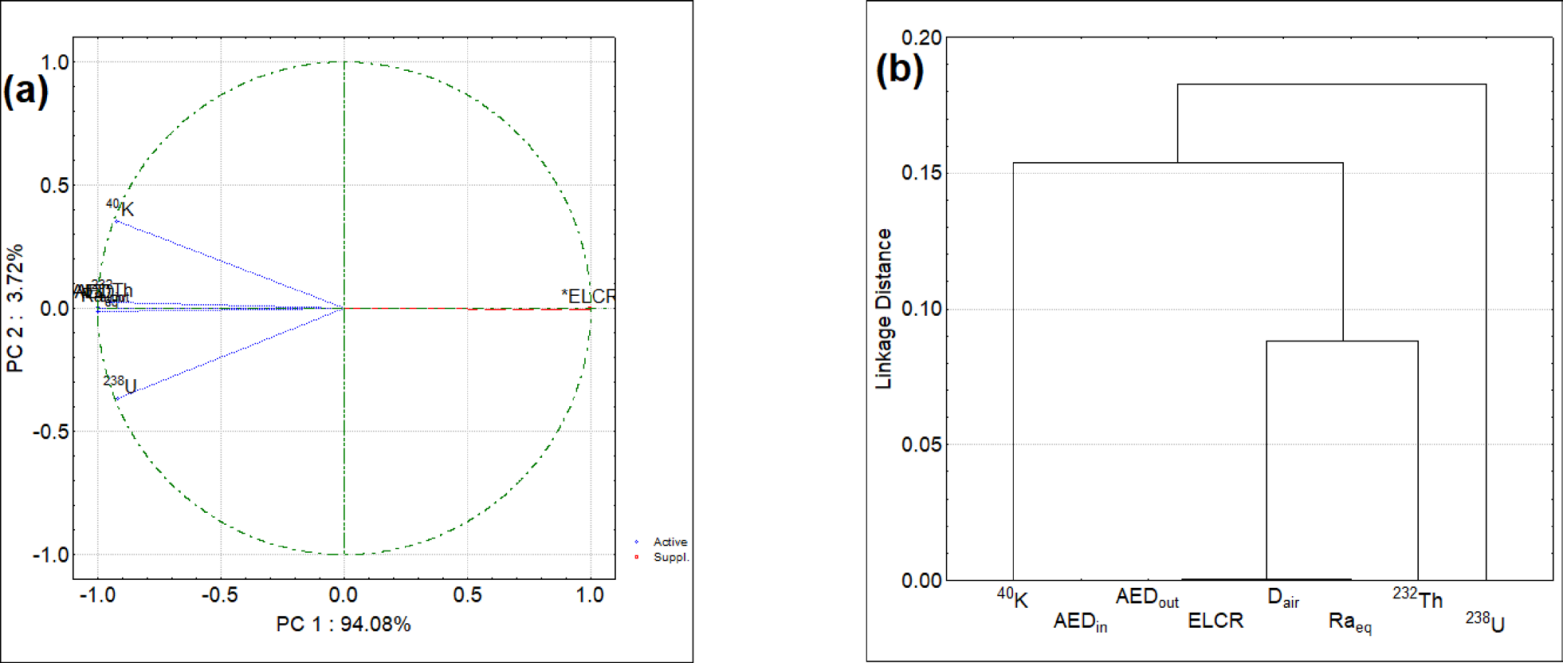
(**a**) The clustering analysis of the radiological parameters and (**b**) Principal component analysis (PC1 and PC2) for radiological data at the studied area, Egypt.

The use of the correlation-based PCA and the Ward’s type HCA for investigating large multi-scale radiological datasets is consistent with previous investigations of environmental radiological datasets. Figure 5 contains two different types of analysis associated with radionuclides ^238^U, ^232^Th, and ^40^K in sedimentary rocks: (Fig. 5a) Principal Component Analysis (PCA) and (Fig. 5b) Hierarchical Cluster Analysis (HCA). The Principal Component Analysis (PCA) and Hierarchical Cluster Analysis (HCA) provide complementary insights into the relationships between radionuclides ^238^U, ^232^Th, and ^40^K in sedimentary rocks. After performing standardization, through the method of Principal Component Analysis (PCA), the first principal component (PC1, 94.08%) represents the overall effect of radiological hazard indices associated with the presence of ^232^Th; Conversely, the second principal component (PC2, 3.72%) captures other variability, mostly associated with ^238^U and ^40^K. The HCA and PCA analyses indicate that there are two interrelated but distinct relationships present in the dataset. One of these relationships is identified by the spatial and statistical properties of both ^232^Th and ^40^K; therefore it can be concluded that they will tend to cluster together based on their respective relationship to lithological and mineralogical controls within their sedimentary depositional environment. Additionally, while the distribution patterns of both ^40^K and ^232^Th are similar from a clustering perspective, there is a greater clustering of the radiological hazard indices (Ra_eq_, D_air_, AED_out_, AED_in_, and ELCR) with respect to ^232^Th than with ^40^K. This difference suggests that the greater radiological contributions that thorium provides than potassium in terms of both dose conversion coefficients and radiological hazard consequences result in ^232^Th being the primary contributor to the radiological hazard parameters while ^40^K is used primarily as a lithologically based cluster and to a lesser extent as a contributory hazard.

### Airborne magnetic data

#### Total magnetic (TM) and reduced to north magnetic pole (RTP)

**Fig. 6:**
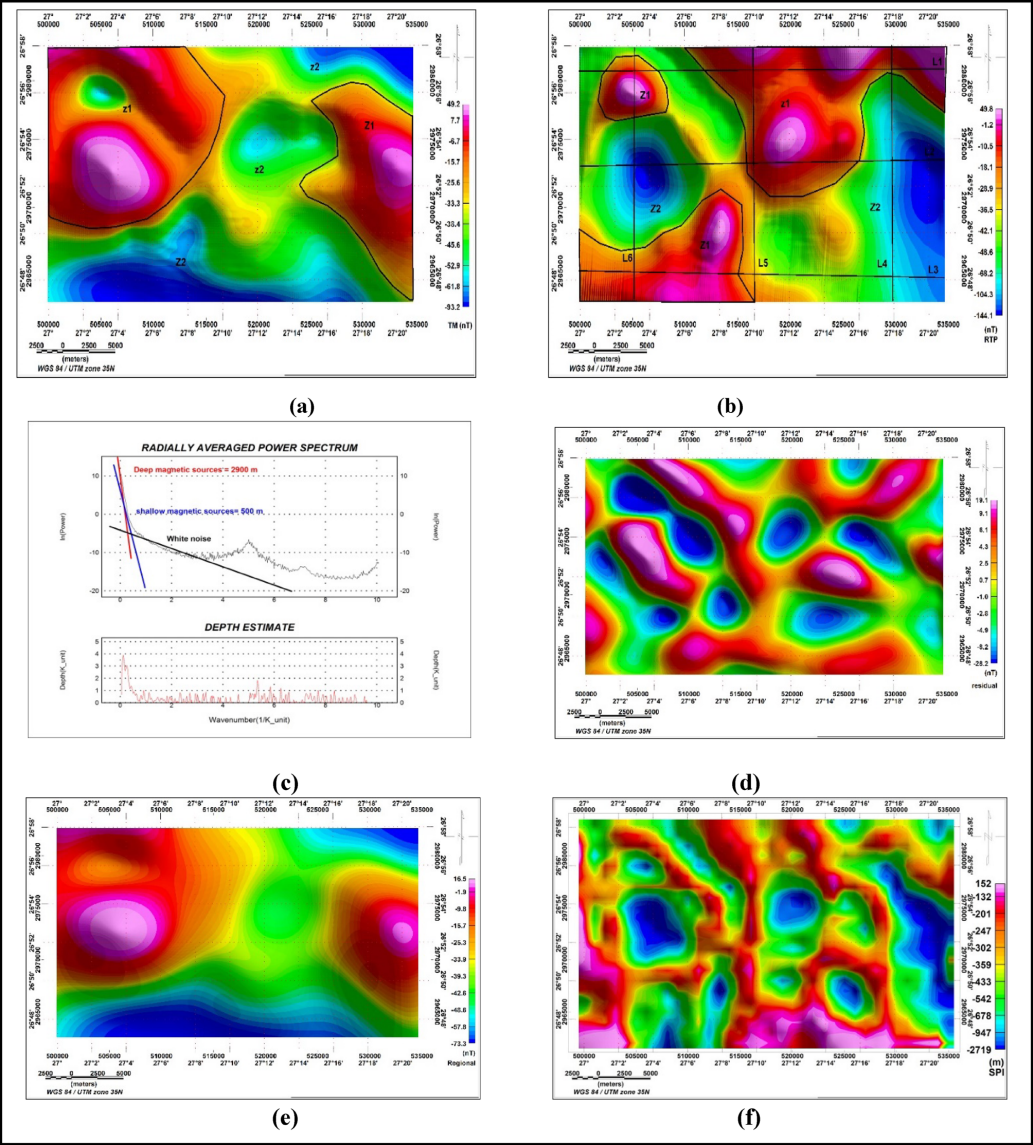
(**a**) Total magnetic map, (**b**) RTP map, ( **c**) Averaged Power Spectrum map, (**d**) Residual map, (**e**) Regional map, (**f**) SPI map west Qar El-Frafra area, Central Western Desert, Egypt.

Figure 6a shows the total-field intensity magnetic map, which indicates the lateral changes in the total intensity of the Earth’s magnetic field. A careful examination of the map revealed that the study area is characterized by the presence of groups of relatively high-positive and low-negative magnetic anomalies of varying sizes and magnitudes. These magnetic anomalies’ fluctuating amplitudes might be a reflection of shifts in the foundation rocks’ composition. Additionally, the differences in the depths of the causative intrusive bodies are shown by the changes in the wavelengths of the magnetic anomalies.

Based on the magnetic characteristics of each zone, two magnetic zones may be identified. The eastern and western regions of the studied area are occupied by the magnetic zone (Z1), which has a positive magnetic character. Additionally, there is a circular positive magnetic anomaly in this zone, which is characterized by a high amplitude and high frequency. The center, southern, and northeastern regions of the prospected area are covered by the magnetic zone (Z2). It is made up of groups of local, typically small-scale, positive magnetic anomalies with low wavelengths and amplitudes that fluctuate in frequency and amplitude. Different lithological compositions or varying relative depths to their causal origins might account for this local variance in the frequency and magnitude of these anomalies. There was a modest variation in magnetic amplitude between the two magnetic zones, which might indicate that the second zone’s causative magnetic entities are mostly somewhat more acidic in composition. The magnetic amplitude gradually diminishes as one moves toward the northern and southern regions of the study region. The comparatively high depths of the causal sources may be reflected in this.

In this region, the total field data were reduced to the pole (RTP) in the frequency domain using an average inclination of 38.6° and declination of 3.2°. Figure 6b shows the RTP aeromagnetic data obtained over the study area. The interpretation of the transformed map is easier, where the anomalies are not shifted as a result of the obliquity of the normal magnetic field. The RTP magnetic map (Fig. 6b) has the same magnetic character, which is illustrated in the total magnetic map (Fig. 6a). Also, the map shows the two magnetic zones, but with a major change in magnetic character at the northern, southern and central parts of the area. Magnetic zone (Z1) occupies the northern, southern and central parts of the investigated area, whereas (Z2) covers eastern and western parts of the study area.

#### Radially averaged power spectrum, residual and regional airborne magnetic maps

A separation filter was applied on the RTP airborne magnetic map, where the regional and residual magnetic-component maps are obtained at 0.088 cycle/K-unit. The estimated average depths for the shallow and deep magnetic sources, as calculated from the power spectrum curve, are 500 m and 2900 m, respectively (Fig. 6c). Residual magnetic anomalies can be defined as the anomalies that are economically interesting because they indicate shallow anomalies and are characterized by weaker and more localized anomalies. The high-pass filtered map (Fig. 6d) of the study area shows a successive system of magnetic dipoles all over the area with various amplitudes and frequencies. The local variation of the frequency and amplitude of these anomalies may be due to the difference in their composition and/or the difference in the relative depth to their sources. The strongest anomalies have E-W, NE-SW and N-S trends, and this part is characterized by elongated positive and negative anomalies of relatively high amplitudes and frequencies. The regional magnetic anomalies are strong, broad, extend over large areas and usually have high amplitudes and low frequencies. These anomalies are of considerable significance in the regional tectonic studies of the basement complex. The false-color image of the RTP magnetic and regional magnetic-component maps (Figs. 6b, e) shows the same number of high and broad magnetic anomalies. These anomalies are characterized by their high magnetic amplitudes and could be discriminated against by the magenta color on the two aeromagnetic maps. The shapes of some of these anomalies are somewhat rounded. These anomalies acquire sharp contacts in various degrees with the surrounding magnetic features. The low-pass filtered map (Fig. 6e) displays an increase in positive magnetic values at the eastern and western parts of the map.

#### Source parameter imaging (SPI)

Source Parameter Imaging (SPI) is a technique based on the extension of complex analytical signals to estimate magnetic depths, it is also known as local wavenumber. The original SPI method^39^ works for two models: a 2-D sloping contact or a 2-D dipping thin-sheet. For the magnetic field M, the local wavenumber is given by:

$$\:k=\:\frac{\frac{{\partial\:}^{2}M}{\partial\:x\partial\:z}\frac{\partial\:M}{\partial\:x}-\frac{{\partial\:}^{2}M}{{\partial\:x}^{2}}\frac{\partial\:M}{\partial\:z}}{{\left(\frac{\partial\:M}{\partial\:x}\right)}^{2}+{\left(\frac{\partial\:M}{\partial\:z}\right)}^{2}}$$

The maximum K for the dipping contact are situated just above the isolated contact edges and is unaffected by the strike, dip, declination, magnetic inclination, and any residual magnetization. The reciprocal of the local wave number is used to measure the depth at the source edge by the equation:

$$\:\boldsymbol{D}\boldsymbol{e}\boldsymbol{p}\boldsymbol{t}{\boldsymbol{h}}_{(\boldsymbol{x}=0)}=\:\frac{1}{{\boldsymbol{K}}_{\boldsymbol{m}\boldsymbol{a}\boldsymbol{x}}}$$

where $\:{K}_{max\:}$is the peak value of the local number K over the step source.

Another benefit of this approach is that, because it makes use of second-order derivatives, the interference of anomalous characteristics is reducible. The technique is applied to gridded data in practice by first predicting the direction at every grid point. The horizontal derivatives are calculated using the least-squares approach in the direction perpendicular to the strike, while the vertical gradient is calculated in the frequency domain.

The massive basement structural height (swell) is marked red on the magnetic depth SPI map (Fig. 6f). This swell extends from the study area’s southwest corner to its northeast corner. The swell’s average depth is 200 m. Two blue-colored subterranean structural lows (troughs) enclose this swell. The trough’s average depth is 2700 m. These depth maps greatly aid us in delineating the overall basement surface features.

#### Forward modeling

An initial model for the source body is created using geology and geophysical intuition in order to forward model magnetic data. After calculating and comparing the model’s anomaly with the observed anomaly, model parameters are changed to enhance the fit between the two anomalies. Until calculated and observed anomalies are judged sufficiently similar, the three-step procedure of body correction, anomaly computation, and anomaly comparison is used.

**Fig. 7:**
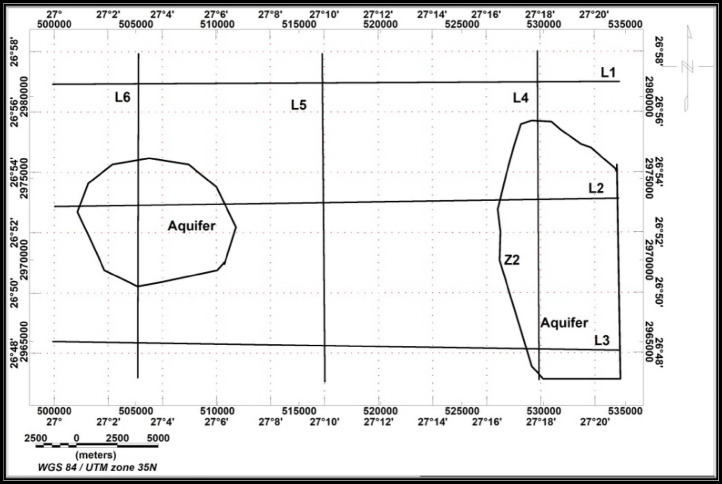
Aquifer extension and location of 2D model, West Qasr El-Frafra area, Central Western Desert, Egypt.

The Geosoft GMSYS program, developed by^40^, based on the techniques of^41^, utilizing procedures was used to carry out the forward modelling. The 2D modelling approach was used to create six magnetic profiles. Various polygons that represented the lithological strata in the research region were given magnetic susceptibility. Since we lacked laboratory observations provided the magnetic susceptibility values for each kind of rock were utilized in the creation of the 2-D forward model. These profiles’ placements on the RTP map as shown in Fig. 7. These profiles were used to track RTP magnetic values. Utilizing the geology data that is already accessible, the magnetic depth measurements that have already been made, and the findings of the qualitative interpretation of magnetic maps. From east to west, three profiles were obtained. Additionally, the remaining profiles were collected from north to south.

**Fig. 8:**
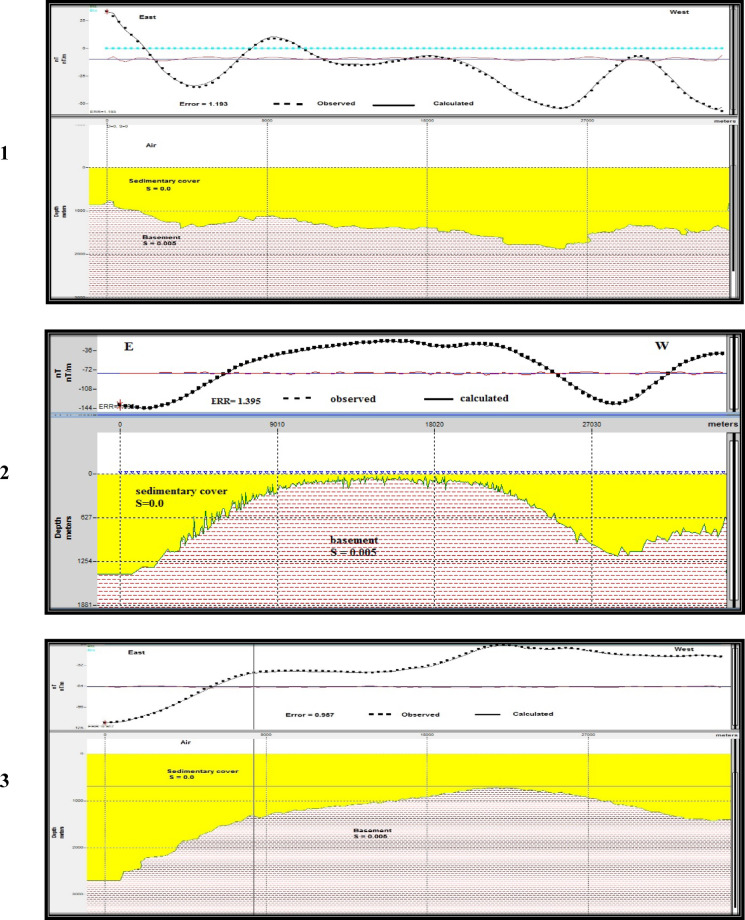
Two-dimensional 2D modelled RTP magnetic profiles 1, 2 and 3, taken from East to West $\:E-W$, West Qasr El-Frafra area, Central Western Desert, Egypt.

**Fig. 9:**
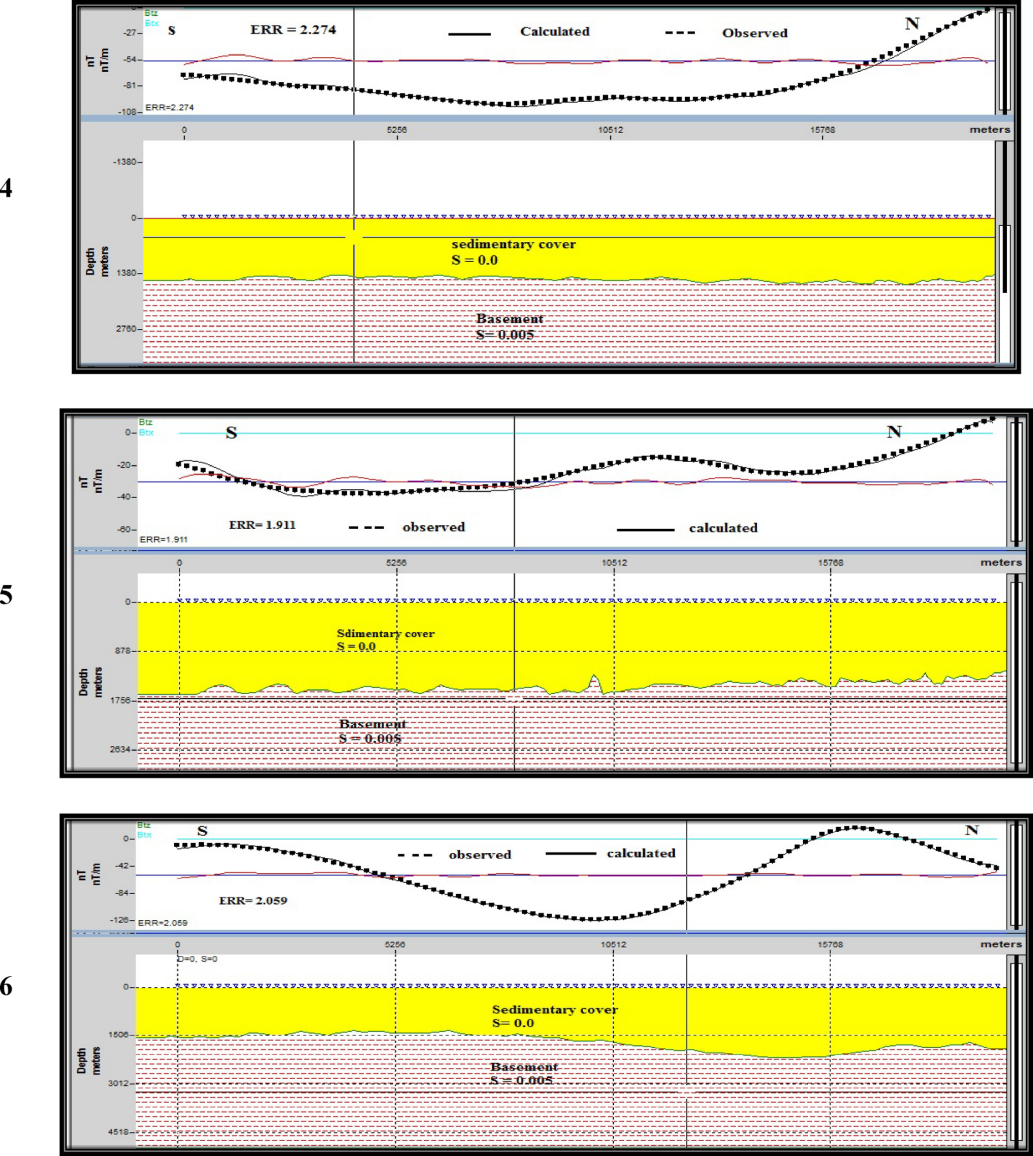
Two-dimensional 2D modelled RTP magnetic profiles 4, 5 and 6, taken from south to north$\:\:\:\:S-N$, West Qasr El-Frafra area, Central Western Desert, Egypt.

The first three profiles that taken from east to west, as illustrated in (Fig. 8), and the second three profiles that taken from south to north, as revealed in (Fig. 9). 2D- models of these profiles confirm that the study area discriminates deeper basement rock, except the central part of the second model of the first three profiles shows shallower basement rocks. Besides, the surface of basement rocks reached more than 1000 m along the hole of all profiles, which is consistent with the averaged depth of the power spectrum SPI depth.

### Integration of magnetic and radiological results

When airborne gamma-ray spectrometry and aeromagnetic datasets are combined, a strong association between surface radiometric trends and how the underlying geology is organized can be established within the study area. Elevated radiometric anomalies tend to occur over structurally moderate to low basement locations as determined from RTP, SPI, and power spectrum analyses. Anomalies tend to occur most frequently in areas corresponding to sediment accumulation, reduced weathering processes, and uranium/thorium-bearing mineral mobilization via groundwater migration through depressions in basement structures. In contrast, basement highs in the northeastern and central portion of the study area tend to correlate with lower radiometric responses, likely resulting from thinner sediment sequences below them and reduced radionuclide-bearing material accumulation. Due to the dominant North-East to South-West (NE-SW), East to West (E-W), and North to South (N-S) magnetic lineations seen in the aeromagnetic datasets, these structural features were determined during the assessment of the datasets to contain sediment following those trends, as well as where radiometric anomalies are located. The analysis of SPI depth data and 2-D magnetic models shows buried basements are more than 1000 m deep in some areas. The deeper basement troughs correlate with several positive radiometric anomalies, indicating structural controls may influence groundwater movement, sediment deposition, and radionuclide distribution. The combined interpretation of radiometric and magnetic datasets provide a better overall understanding of the geology, structure, radiation in the environment, and hydrogeological potential, of the West Qasr El-Farafra area. Integrated interpretation indicates that radionuclide distributions are influenced by factors other than changes in lithology; they also depend on subsurface structural features identified through aeromagnetic analysis. Basement depressions appear to aid in the accumulation of sediments and groundwater movement, leading to localized concentrations of uranium and thorium. Therefore, combined use of airborne gamma-ray spectrometry and aeromagnetic data is a better basis for assessing radiation levels in the environment than either type of data alone, and provides additional information on how geological factors affect the distribution of radionuclides in the study area.

## Conclusion

A comprehensive regional assessment of natural radon nuclides and associated radiation exposure in the West Qasr El-Farafra area has been made utilizing high-density airborne gamma ray spectrometry and magnetic data. Moreover, the findings of the study offer a reliable regional radiological baseline to enable evaluation of environmental safety and inform future urban and agricultural planning in the West Qasr El-Farafra region. Average activity of ^238^U (37 ± 18 Bq kg^− 1^) and ^232^Th (32 ± 14 Bq kg^− 1^) have activity levels that are comparable or lower than world averages, while the average activity of ^40^K (665 ± 205 Bq kg^− 1^) is higher than the world average due to the abundance of potassium-bearing minerals. The radiological hazard index values (Ra, absorbed dose rate, and annual effective dose) generally fall within the recommended international limits; however, elevated localized values for these indices indicate they need to continue to be monitored. It was determined through multivariate analyses that ^232^Th is the most significant contributor to the dose-related parameters, while the additional presence of magnetic data allows an overview of the local geology and basement structure, which assists in determining basement depths and subsurface contours. The surface of basement rocks reached more than 1000 m along the hole of all areas, depending on 2-D magnetic modelling profiles, which is consistent with the averaged depth of power spectrum and SPI depths, and the area has a good aquifer. The combination of radiological and magnetic data establishes an environmental radiation baseline for the area and supports regional land use planning and initial assessments of public exposure. As a result, it was concluded that while the area can be developed from a radiological perspective, management of localized anomalies and site-specific investigations should take place as needed to mitigate radiological hazards from development activities.

## Data Availability

The datasets used and/or analyzed during the current study available from the corresponding author on reasonable request.

## Code availability

No custom code, novel algorithm, or proprietary computational pipeline was developed for this study and all processing and analysis were done using existing software packages: geophysical data were processed using Oasis Montaj, statistics were calculated using SPSS, and magnetic forward modelling was completed using Geosoft GM-SYS. Therefore, there is no code available for deposition.
